# Exploration the global single-cell ecological landscape of adenomyosis-related cell clusters by single-cell RNA sequencing

**DOI:** 10.3389/fgene.2022.1020757

**Published:** 2022-10-17

**Authors:** Jiajing Lin, Li Liu, Fengque Zheng, Saiqiong Chen, Weiwei Yang, Jingjing Li, Steven Mo, Ding-Yuan Zeng

**Affiliations:** ^1^ Department of Obstetrics and Gynecology, No. 4th Hospital Affiliated to Guangxi Medical University, Liuzhou, China; ^2^ Department of Gynecology, Liuzhou Maternity and Child Healthcare Hospital, Liuzhou, China; ^3^ Department of Basic Science, YuanDong International Academy of Life Sciences, Hong Kong, China

**Keywords:** adenomyosis, single-cell RNA sequencing, malignant cells, immune microenvironment, biological function

## Abstract

**Background:** Adenomyosis (AM) is a common benign uterine disease that threatens the normal life of patients. Cells associated with microenvironmental immune ecology are crucial in AM, although they are not as well understood at the cellular level.

**Methods:** Single-cell sequencing (scRNA-seq) data were used to construct an AM global single-cell map, to further identify relevant cell clusters and infer chromosomal copy number variation (CNV) in AM samples. The biological functions of cell clusters were explored and cellular evolutionary processes were inferred by enrichment analysis and pseudotime analysis. In addition, a gene regulatory network (GRN) analysis was constructed to explore the regulatory role of transcription factors in AM progression.

**Results:** We obtained the expression profiles of 42260 cells and identified 10 cell clusters. By comparing the differences in cell components between AM patients and controls, we found that significant abundance of endometrial cells (EC), epithelial cells (Ep), endothelial cells (En), and smooth muscle cells (SMC) in AM patients. Cell clusters with high CNV levels possessing tumour-like features existed in the ectopic endometrium samples. Moreover, the Ep clusters were significantly involved in leukocyte transendothelial cell migration and apoptosis, suggesting an association with cell apoptosis and migration. En clusters were mainly involved in pathways in cancer and apoptosis, indicating that En has certain malignant features.

**Conclusion:** This study identified cell clusters with immune-related features, investigated the changes in the immune ecology of the microenvironment of these cells during AM, and provided a new strategy for the treatment of AM.

## Introduction

Adenomyosis (AM) is a common benign gynecological syndrome, characterized by infiltration of endometrial glands and stroma in the myometrium (Bird et al., 1972). The prevalence of AM ranges from 5% to 70% (Graziano et al., 2015), with an average of 20%–35% of women worldwide suffering from AM (Abbott, 2017). The most common manifestations of AM are dysmenorrhea, infertility, and abnormal uterine bleeding (AUB), but some women with AM are asymptomatic (Peric and Fraser, 2006). From the epidemiologic data, AM can increase the risk of cancers, including ovarian, endometrial, breast, colorectal, and other cancers of women (Borgfeldt and Andolf, 2004; Kok et al., 2015). Furthermore, Bergeron previously reported that the definitive diagnosis of AM was based on the presence of ectopic endometrial tissue in the myometrium (Bergeron et al., 2006), but is now diagnosed by non-invasive techniques such as pelvic imaging (Chapron et al., 2020). Studies have also reported that the standard method of managing the disease is hysterectomy, while most patients desire to preserve their fertility (Stratopoulou et al., 2021). Despite improvements in diagnostic tools, awareness of AM remains poor (Leyendecker et al., 2015).

At the cellular level, the microenvironmental immune cells of AM play an important role. The number of macrophages, natural killer cells, and T cells in the endometrial stroma of AM increased significantly compared with women with mild focal AM or without the disease (Yang et al., 2004; Tremellen and Russell, 2012). Several malignant features exist in the epithelial cells (Ep) of AM, such as high migration capacity, which contribute to disease progression (Liu et al., 2021). Studies have confirmed that the endothelial cells (En) are damaged, and the uterus occurs the symptoms of bleeding, which is also important in adhesion and migration (Kruger-Genge et al., 2019). Furthermore, smooth muscle cells (SMC) have the ability to shrink and diastole, lack of contraction can cause uterine bleeding, which may lead to the occurrence of inflammation (Owens et al., 2004). It may thus be possible to comprehend the emergence of AM by concentrating on the mechanisms of change in cells related to the immunological microenvironment.

Single-cell RNA sequencing (scRNA-seq), an indispensable technique to dissect cellular heterogeneity and analyze cell types, can assist us in thoroughly comprehending the biological roles (Hedlund and Deng, 2018). Numerous effective methods to examine molecular alterations at the cellular level are provided by the scRNA-seq (Tang et al., 2009). Moreover, research has demonstrated that rare clusters of AM were identified by scRNA-seq, confirming that the occurrence originates from endometrial migration (Liu et al., 2021). However, more studies are needed for further validation. In this study, we explored the states and transitions of the immune microenvironment cells of AM from a single-cell perspective. A comprehensive map of the AM single-cell ecosystem was depicted, relevant cell clusters were identified, and chromosomal copy number variation (CNV) was inferred for each AM sample. Furthermore, it was further confirmed the associated cluster of markers was involved in the signaling pathways and gene regulatory networks (GRN), which contributes to our understanding of the functions of the cluster markers in AM and at the cellular level.

## Materials and methods

### Data sources

The AM scRNA-seq data including SRR12791871, SRR12791872, and SRR12791873 (Liu et al., 2021) were obtained from the Sequence Read Archive (SRA) of the National Center for Biotechnology Information (NCBI). A 50-year-old woman with uterine fibroids, excluding the AM, and the endometrium tissue from this patient were used as a control sample. Moreover, two endometrium tissue samples were obtained from a 46-year-old AM patient and the samples were taken from eutopic endometrium (AM_EM) and ectopic endometrial (AM_EC) tissues.

### Data preprocessing and construction of the single-cell atlas

We used the IntegrateData function (Butler et al., 2018) in the Seurat package (Stuart et al., 2019) to merge the scRNA-seq data, and performed cell clustering analysis according to default parameters. Uniform Manifold Approximation and Projection (UMAP) algorithm (Becht et al., 2018) was adopted for dimensionality reduction and visualization and mapped into single cell profiling. Subsequently, the FindAllMarkers function in Seurat package identified the specific marker genes in each cell cluster. Furthermore, the cell types underwent an immune response based on annotation and re-clustering of known marker genes.

### Differential gene expression analysis

Differential expression analysis was performed based on the FindMarkers function in the Seurat package (Butler et al., 2018). Differentially expressed genes (DEGs) of different clusters in the Control, AM_EM, or AM_EC groups were identified. DEGs between normal tissues and AM tissues were screened by an adjusted *p* value <0.05 and |log fold change (log FC)|>0.5 being considered significant.

### Evaluation of CNV in single cells

CNVs are primarily used to identify subclones of diseased cells and to infer tumor evolution (Yates and Campbell, 2012). The CNVs of each cell were assessed from the AM patients by the inferCNV package (inferCNV of the Trinity CTAT Project; https://github.com/broadinstitute/inferCNV) (Patel et al., 2014). To calculate the CNVs of AM_EM and AM_EC cells, the average or normal expression of genes from immune cells was applied as a reference and then determine the expression.

### Functional enrichment analysis

To explore the biological functions involved in each cell cluster. We performed the Gene Ontology (GO) and Kyoto Encyclopedia of Genes and Genomes (KEGG) on the clustered markers using the clusterProfiler package (Yu et al., 2012). *p* < 0.05 was considered statistically significant.

### Pseudotime analysis

Pseudotime analysis could determine the dynamics of gene expression within cell types and trajectories over time (Cao et al., 2019), and infer cell evolution during the AM. A “branch” trajectory was constructed based on the Monocle three package (Trapnell et al., 2014) to explore the dysregulated changes in immune cells of AM patients and project the cells into low-dimensional space by UMAP, the parameters of Monocle three package are set to the default.

### Construction of GRN

In this study, we constructed a GRN with transcription factors as the core to infer co-expression modules, to further explore the dysregulate regulatory mechanism of immune cellogenesis. Single cell regulatory network inference and clustering (SCENIC) (Aibar et al., 2017; Van de Sande et al., 2020) was used to infer gene regulatory networks based on single-cell expression profiles and identify cell states, providing important biological insights into the mechanism driving cell heterogeneity. Among them, the binding motifs of the transcription factors in the co-expression module were obtained from the JASPAR database (https://jaspar.genereg.net).

### Statistical analysis

Statistical analyses were performed using R (https://www.r-project.org/). Expression levels of genes were analyzed using unpaired t-tests. If the *p* < 0.05 that considered statistical significance. The analyses in this study were based on the Bioinforcloud platform (http://www.bioinforcloud.org.cn).

## Results

### Global single-cell atlas of the adenomyosis

**FIGURE 1:**
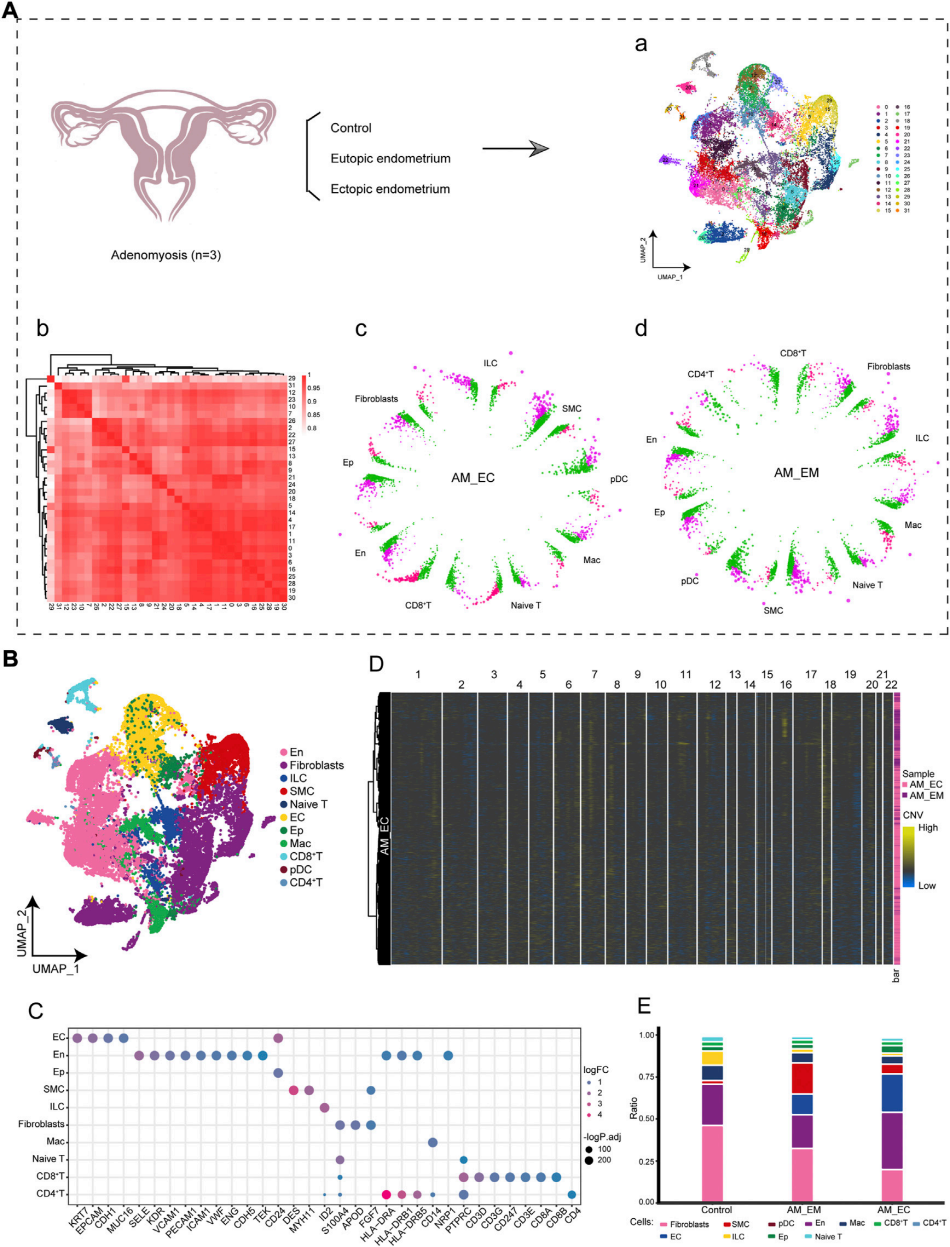
Single-cell landscape of adenomyosis (AM) in the immune microenvironment. **(A)**. The workflow of this study analyzed single-cell RNA sequencing (10X Genomics) data between control and two AM between eutopic endometrium (AM_EM) and ectopic endometrium (AM_EC) samples, identifying 31 cell clusters of 42,260 cells. **(B).** The cell types of single cells mapping AM includes endometrial cells (EC), fibroblasts (Fibroblasts), epithelial cells (Ep), endothelial cells (En), CD8 + T, CD4 + T, initial T cells (Naive T), macrophages (Mac), predendritic cells (pDC), smooth muscle cells (SMC), and innate lymphoid cells (ILC). **(C)**. Bubble plots show specific marker genes in different cell types. **(D).** Heatmap shows the levels of copy number variation in chromosomes 1 to 22 during AM. **(E).** Differences in cell abundance components between control and AM patients. Different colors represent different cell types. AM_EM, eutopic endometrium; AM_EC, ectopic endometrium; CNV, copy number variations; EC, endometrial cells; Ep, epithelial cells; En, endothelial cells; Naive T, initial T cells; Mac, macrophages, pDC, predendritic cells; SMC, smooth muscle cells; ILC, innate lymphoid cells; UMAP, Uniform Manifold Approximation and Projection.

To investigate the early cell population dynamics in AM patients, we analyzed the scRNA-seq data from endometrial tissue samples of AM patients and control donors. The analysis flow of this study is shown in Figure 1A, where we constructed a global single-cell landscape of AM. By cluster analysis, we divided 42,260 cells into 31 cell clusters, which were identified into 11 cell types based on known markers (Figure 1B) (Supplementary Table S1), including endometrial cells (EC), fibroblasts, epithelial cells (Ep), endothelial cells (En), CD8^+^T, CD4^+^T, Naive T, macrophages (Mac), plasmacytoid predendritic cells (pDC), smooth muscle cells (SMC), and innate lymphoid cells (ILC). Among them, each cell marker exhibited a specific expression for the cell cluster (Figure 1C). Chromosomal CNV analysis based on expression patterns at genomic intervals showed the presence of multicopy events in AM in ectopic endometrial samples (Figure 1D). Further comparing the differences in cell composition between control and AM patients, we found that the highest abundance of En and EC was found in the AM_EC and AM_EM groups. However, the fibroblasts in the control group had the highest abundance (Figure 1E). In summary, we delineated the single-cell profiles of AM patients to reveal the differences in microenvironmental cell components in AM patients.

### Ecological landscape of adenomyosis-associated endometrial cells clusters

**FIGURE 2:**
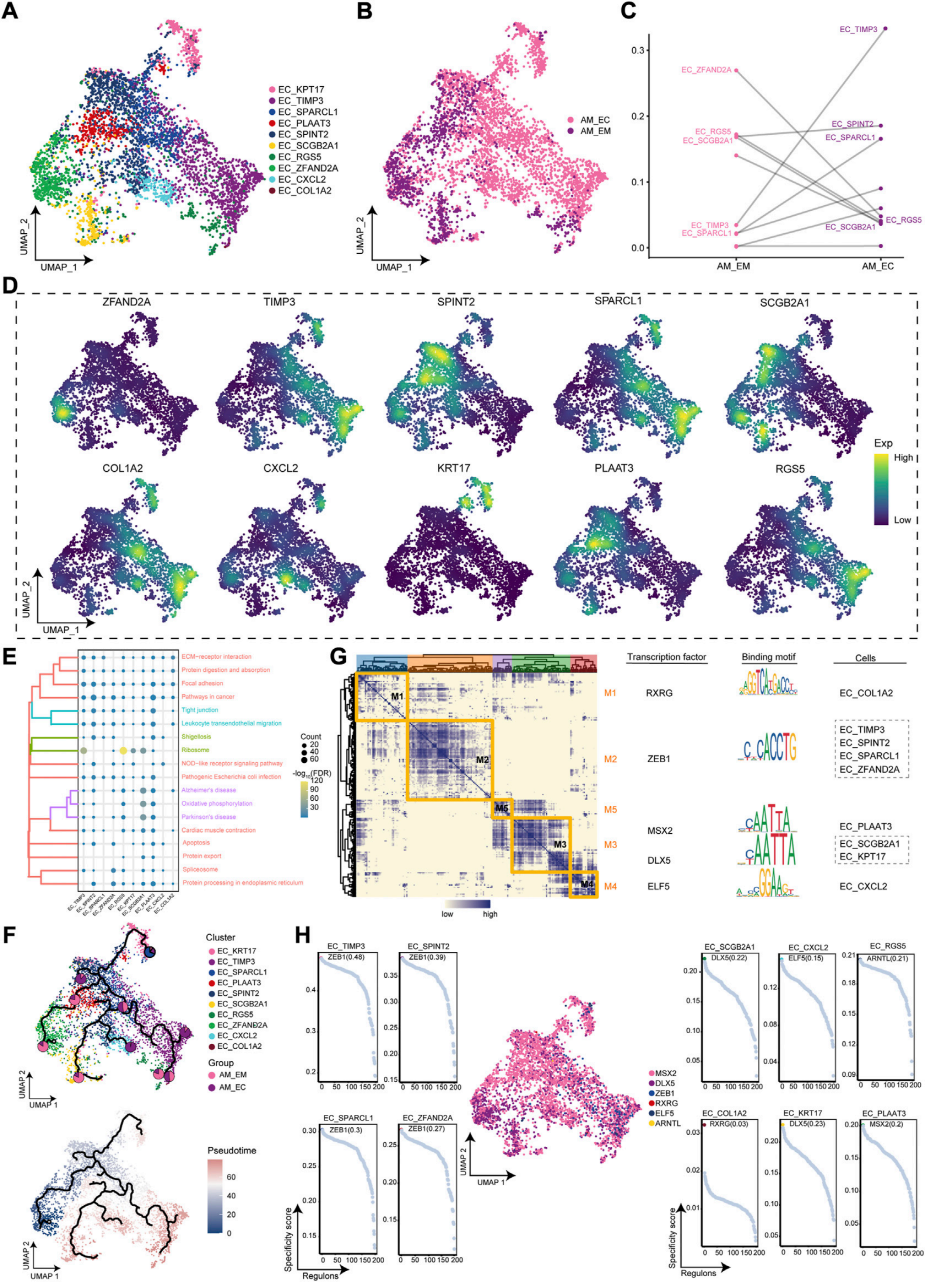
Identification of the endometrial cells (EC) clusters in adenomyosis (AM). **(A)**. Single-cell atlas shows the cellular clusters of EC. **(B).** Single-cell atlas shows clusters of EC cells in eutopic endometrium and ectopic endometrial samples from AM patients. **(C)**. Differences in the abundance of EC clusters in the eutopic and ectopic endometrium groups of AM patients. **(D).** Marker genes for the specific EC clusters. **(E).** Biological pathways in specific clusters of EC. **(F).** Single-cell atlas map the trajectory and pseudotime values of EC progression. Pie charts show the proportion of the different groups in the clusters. **(G).** Heatmap-TF-binding sequence-cell clusters demonstrate the gene regulatory network of EC clusters. **(H).** Transcription factors regulate the makers of EC clusters. AM_EM, eutopic endometrium; AM_EC, ectopic endometrium; EC, endometrial cells; UMAP, Uniform Manifold Approximation and Projection.

AM occurs mainly in endometrial tissue, which has a higher cell abundance in EC of AM patients, therefore, subsequent studies will focus on this cellular cluster. We obtained 10 EC clusters by cluster analysis (Figure 2A). As shown in Figure 2B, almost all of these cell clusters were present in different groups of AM patients. Further exploration of the abundance of the cellular cluster in AM patients revealed a significant increase in the proportion of EC_TIMP3 cell clusters and a significant decrease in the proportion of EC_ZFAND2A clusters (Figure 2C). Markers for the different clusters of the EC were mapped to the single-cell atlas, including ZFAND2A, KPT17, TIMP3, SPARCL1, PLAAT3, SPINT2, SCGB2A1, RGS5, CXCL2, and COL1A2 (Figure 2D). Furthermore, EC clusters may be associated with cell motility, which was closely associated with focal adhesion and leukocyte transendothelial migration and apoptosis (Figure 2E). Based on the pseudotime trajectory analysis, it was inferred that the EC_ZFAND2A cluster served as the developmental starting point, and then differentiated into other cell clusters (Figure 2F). Furthermore, we constructed the GRN and found that the GRN with TFs as pivots was organized into five modules (Figure 2G), such as RXRG, ZEB1, MSX2, DLX5, ELF5, ARNTL, to regulate the specific gene expression (Figure 2H). Above all, we identified EC clusters of AM patients, defined marker genes for their specific expression, and elucidated the GRN of evolved EC clusters.

### Ecological landscape of adenomyosis-associated en clusters

**FIGURE 3:**
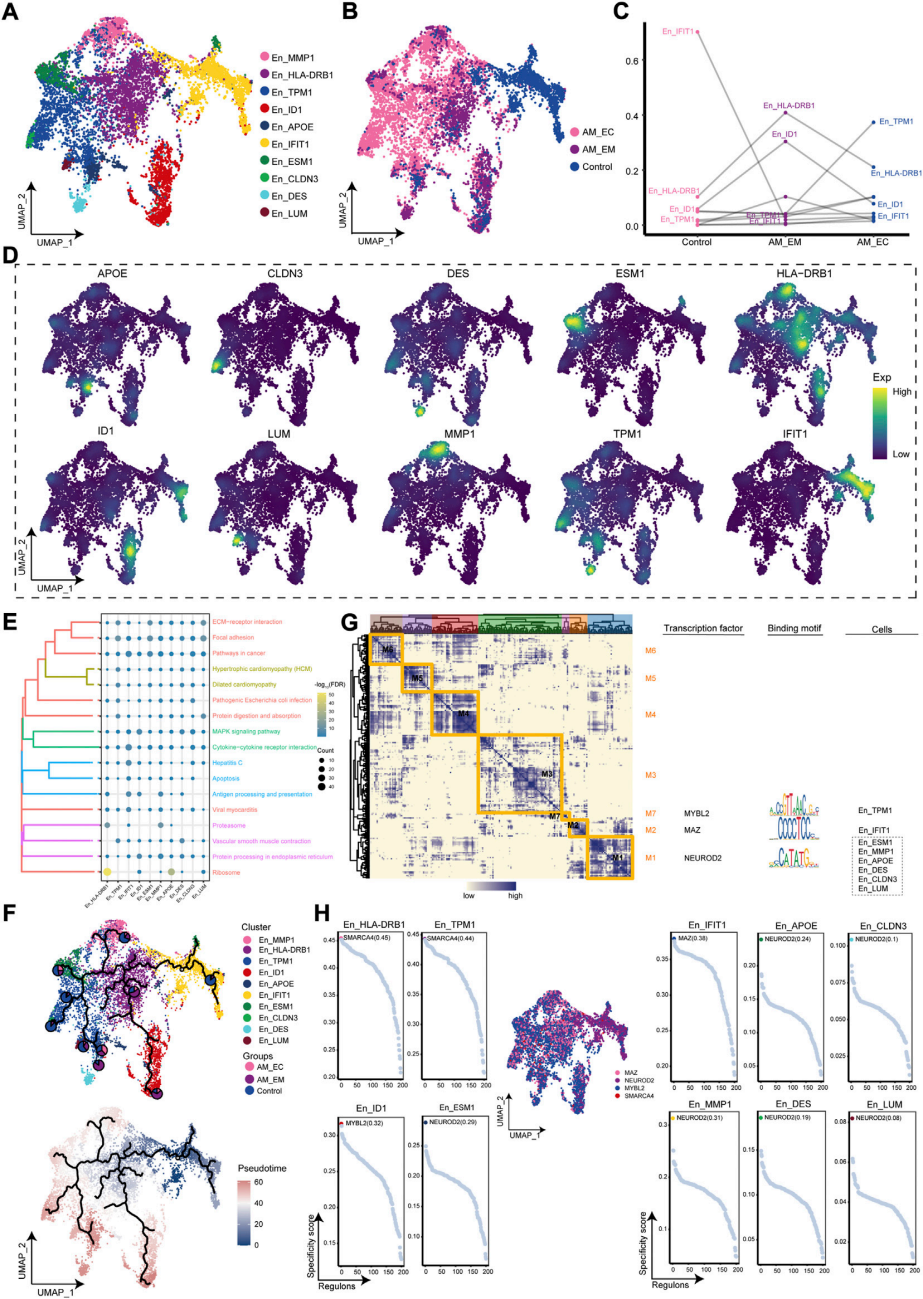
The adenomyosis (AM)-associated endothelial cell (En) clusters. **(A)**. Single-cell atlas shows the cell clusters of endothelial cells (En). **(B).** Single-cell mapped the En clusters in the eutopic endometrium (AM_EM), ectopic endometrial (AM_EC), and control groups of AM patients. **(C).** Differences in the abundance of En clusters among the AM_EM, AM_EC, and control groups. **(D).** Marker genes for specific En clusters. **(E).** Biological pathways in specific En clusters. **(F).** Single-cell atlas map the trajectory and pseudotime values of development in En from AM. Pie charts show the proportion of the different groups in the clusters. **(G).** Transcription factors of En clusters in a co-expression pattern. Left: Heat map identifies co-expression modules; middle: major transcription factors and binding sequences; right: cell clusters of transcription factors. **(H).** Transcription factors regulated makers of EC clusters. AM_EM, eutopic endometrium; AM_EC, ectopic endometrium; UMAP, Uniform Manifold Approximation and Projection.

The abundance of En was significant in the AM_EC group, from which we inferred that En may play a facilitating role in the disease course. Therefore, the cluster analysis of En yielded 10 cell clusters (Figure 3A). Further mapping of these cell clusters to the AM_EC, AM_EM, and control groups, and we found that mainly originated from AM patients (Figure 3B). En_HLA-DRB1 and En_ID1 were increased in AM_EM, En_TPM1 was significant in the AM_EC group, and En_IFIT1 was significantly decreased in AM patients (Figure 3C). Gene expression of markers for En clusters weas mapped to a single-cell atlas, including APOE, CLDN3, MMP1, HLA-DRB1, TPM1, ID1, IFIT1, ESM1, DES, and LUM (Figure 3D). En clusters were mainly related to cell proliferation, such as pathways in cancer, apoptosis and extracellular matrix receptor interactions (Figure 3E). Pseudotime trajectory analysis showed that the En_IFIT1 cluster evolved as a developmental starting point towards AM patients (Figure 3F). The results of GRN for En clusters indicated that marker genes were divided into seven modules, and regulated by TFs, such as MYBL2, MAZ, and NEUROD2 (Figure 3G). Figure 3H shows the transcription factors of En specific cell cluster. En cluster markers are regulated by transcription factors that promote the development of AM.

### Exploring the ecological landscape in ep clusters of adenomyosis

**FIGURE 4:**
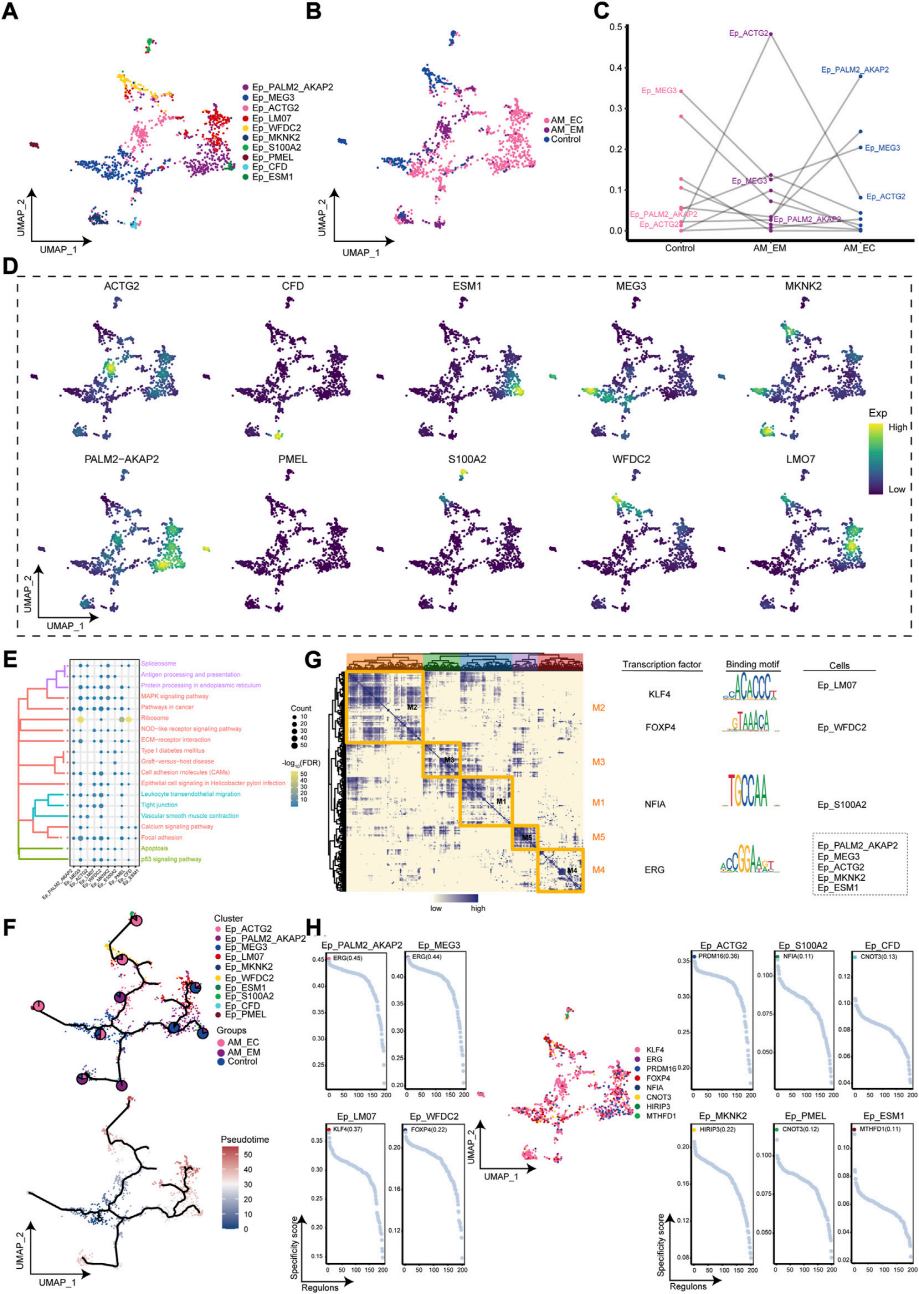
The adenomyosis (AM)-associated epithelial cell (Ep) clusters. **(A)**. Single-cell mapped of cell clusters of Ep. **(B).** Single-cell atlas shows the AM_EM, AM_EC, and control groups with clusters of Ep. **(C).** Differences in the cellular ecology of Ep clusters in different groups. **(D).** Marker genes for specific Ep clusters. **(E).** Biological pathways in Ep clusters. **(F).** Single-cell atlas map the developmental trajectory of Ep in AM. Pie charts show the proportion of the different groups in the cluster. **(G).** Transcription factors of Ep clusters in a co-expression pattern. Left: Heat map identified co-expression modules; Middle: major transcription factors and their binding sequences; Right: cell clusters of transcription factors. **(H).** Scatter plot of transcription factors in Ep clusters. AM_EM, eutopic endometrium; AM_EC, ectopic endometrium; UMAP, Uniform Manifold Approximation and Projection.

Interestingly, Ep loses polarity and intercellular adhesion to gain migration capacity (Acloque et al., 2009), and the cell abundance of Ep clusters was significant in the AM_EC group. Therefore, cluster analysis of Ep clusters was again performed to obtain 10 cell clusters (Figure 4A), which were mapped to AM_EC, AM_EM, and control groups according to their sample source (Figure 4B). Compared with the control group, the cell abundance of Ep_ACTG2 was significantly increased in the AM_EM group, cell abundance of Ep_PALM2_AKAP2 was significant in the AM_EC group (Figure 4C). Subsequently, we mapped the expression of cluster markers (PALM2_AKAP2, MEG3, ACTG2, LM07, WFDC2, MKNK2, S100A2, PMEL, CFD, and ESM1) to the single-cell atlas of Ep clusters (Figure 4D). To further explore the biological signatures for the involvement of the Ep clusters in the AM, we performed enrichment analysis of the marker genes in the Ep clusters, showing that extracellular matrix receptor interactions, MAPK signaling pathway and apoptosis were significantly involved in Ep clusters (Figure 4E). The developmental trajectory of Ep was explored by pseudotime trajectory analysis, and the results indicated Ep_PMEL cluster was in an early developmental stage and evolved into AM_MKNK2, AM_ESM1, AM_LM07, and AM_S100A2 (Figure 4F). We further performed a GRN analysis of Ep clusters, showing that the marker genes of the Ep clusters were divided into five modules regulated by the transcription factors, such as KLF4, FOXP4, NFIA, and ERG (Figure 4G). Furthermore, we explored the expression of these TFs in specific Ep clusters and found that KLF4 was the most highly expressed in the cluster (Figure 4H), suggesting that high expression of KLF4 may be associated with the development of AM.

### SMC-associated cell clusters of adenomyosis

**FIGURE 5:**
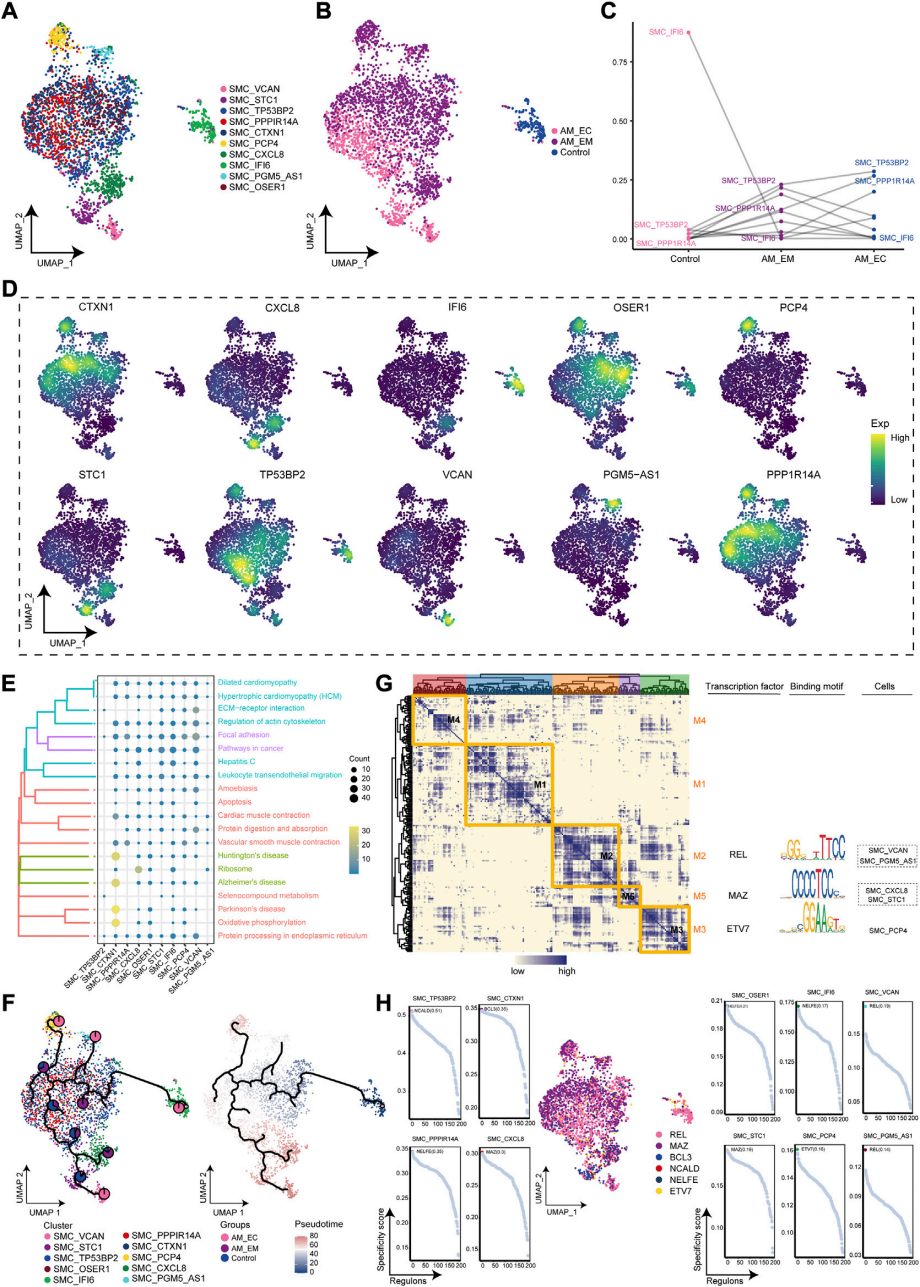
AM-related smooth muscle cell (SMC) clusters **(A)**. Single-cell atlas maps the cell clusters of SMC. **(B).** Single-cell atlas shows different groups of SMC clusters. **(C).** Differences in the cellular ecology of SMC clusters in different groups. **(D).** Marker genes for specific SMC clusters. **(E).** Biological pathways are significantly involved in the SMC clusters. **(F).** Single-cell atlas maps the evolutionary trajectories of SMC subgroups in AM. Pie charts show the proportion of the different groups in the subgroup. **(G)**. Transcription factors for clusters of SMC in a co-expression pattern. Left: Heat map identifies co-expression modules; middle: major transcription factors and their binding sequences; right: cell clusters of transcription factors. **(H).** Scatter plots showed transcription factors in clusters of SMC. AM_EM, eutopic endometrium; AM_EC, ectopic endometrium; SMC, smooth muscle cell; UMAP, Uniform Manifold Approximation and Projection.

During AM, ecological components of SMC clusters were significantly observed, so we will investigate their microenvironmental immune properties. The SMC clusters were re-clustered to obtain 10 cell clusters (Figure 5A) and mapped to different samples (Figure 5B). The SMC_TP53BP2 cluster had significant cellular abundance in the AM_EC and AM_EM groups compared to the control group, while SMC_IFI6 had significant cellular abundance in the control group (Figure 5C). Next, we showed the expression of marker genes was significantly changed in SMC clusters for clusters (Figure 5D). The SMC clusters were significantly involved in pathways, such as cytokine receptor interaction, vascular smooth muscle contraction and apoptosis (Figure 5E). We also further explored the developmental trajectory of the SMC, clarifying the evolution trajectory from the SMC_TP53BP2 cluster to the SMC_IFI6, SMC_VCAN, and the SMC_CXCL8 cluster (Figure 5F). In addition, the GRN analysis with TFs as the fulcrum yielded five modules (Figure F5G) with gene expression of specific SMC regulated by REL, MAZ, and ETV7 (Figure 5H). Taken together, these results suggest that certain specific clusters are closely associated with vascular smooth muscle contraction, can lead to smooth muscle ischemia in AM patients, and may promote the development of AM.

## Discussion

To date, most studies of orthoendometrium in women with AM have focused on the expression of a single gene or a limited number of genes (Benagiano and Brosens, 2012; Xiaoyu et al., 2014). However, few studies have revealed the uniqueness of each cell in the AM process at the individual cellular level. In this study, we analyzed the scRNA-seq data from the endometrial tissue of one patient with AM and one fibroid patient as controls, and explored changes in the cellular state and immune microenvironment of AM. Ectopic endometrium samples present high CNV levels, and are considered a potential factor in the development of the AM. The presence of certain specific cell clusters was associated with the progression of AM. In conclusion, it is possible to explore the global status of AM patients at the cellular level, providing new insights into the in-depth study of AM.

The ecological compositions of EC in the AM_EC and AM_EM groups were significant compared to the controls, indicating that EC plays an important role in the AM microenvironment. EC clusters were significantly involved in cell motility-related pathways such as focal adhesion and leukocyte transendothelial migration by enrichment analysis. AM patients suffer from uterine bleeding, pelvic pain, or infertility in women due to endometrial adhesion and destruction of the endometrium (Lacheta, 2019; Bulun et al., 2021). In the GRN, RXRG, ZEB1, MSX2, DLX5, and ELF5 regulated the EC clusters. Knockdown of SKP2 was found to reduce ZEB1 expression in endometrial stromal cells, thereby inhibiting their invasion and proliferation (Guo et al., 2022). However, few other TFs have been reported in AM. although many relevant mechanisms have been studied based on endometrial tissue, few studies have determined the effect of EC cells on AM at the cellular level. In conclusion, these results suggested that EC cells mediate and disrupt the endometrium, moreover, a cluster of markers regulated by TFs may promote the development of AM.

En has a dual role in immunology and pathology. On the one hand, the dysfunction will mediate the development of certain diseases, and on the other hand, they will actively mediate the immune response at the site of injury or infection (Pober and Sessa, 2007). Liu found co-localization of Ep and En markers in cluster one and promoted cell growth in AM (Liu et al., 2021), identical to the tumor-like characteristics reported by AM (Liu et al., 2018). Enrichment analysis showed that the En clusters were significantly involved in cancer-related pathways and extracellular matrix receptor interactions, indicating that the En was associated with cell proliferation and had certain malignant characteristics in the AM_EC group. Moreover, KLF4, FOXP4, NFIA, and ERG can regulate the markers of En clusters in the GRN. By inhibiting the biological functions of autophagy and metaphase during AM onset, KLF4 is abnormally reduced (Mei et al., 2022). However, other regulated TFs were almost rarely reported in AM, and whether En migrate needs further investigation.

Ep recognizes perturbations in their microenvironment, sends reinforcement signals, and transmits the signals to the immune system (Larsen et al., 2020). Studies have shown that epithelial immune cells of endometria can enhance cell survival and epithelial protective barrier function (Ho et al., 2006). Notably, the highest percentage of EP was found in the AM_EC and AM_EM groups; however, the number of EP was lower in the control group, which may be due to the significant postoperative endometrial thinning in the control group. Moreover, the Ep clusters were also significantly involved in the biological pathways such as leukocyte transendothelial migration, the MAPK pathway, and focal adhesions. Migration across the En after leukocyte adhesion, indicated that Ep was associated with high motility and migration. The MAPK pathway was required for the cell migration process, and the cytokines can also mediate cell migration (Liu et al., 2021). Furthermore, development of AM was improved by inhibiting the activated MAPK/ERK signaling pathway (Ying et al., 2021). In addition, study has shown that Ep loses their polarity and intercellular adhesion during adhesive epithelial interstitial transformation (EMT), to gain the ability to migrate to a mesenchymal phenotype (Acloque et al., 2009) and that EMT may play a key role in the pathogenesis of AM (Wang et al., 2021). In particular, during the conversion of Ep into En, a significant accumulation of angiogenic mimicry formation in AM_EC was found (Liu et al., 2021). Our results suggested that EP subsets significantly involved in pro-migratory pathways may play an important role in AM progression.

Abnormal proliferation of SMC in the endometrium-myometrial junction area is an important cause of AM (Huang et al., 2021), and the emergence of AM causes hyperplasia and hypertrophy of the surrounding SMC (Zhai et al., 2020). Compared to the SMC in the normal uterus, the uterine SMC has hypertrophy and ultrastructural changes, which may have contractility effects on the myometrium (Mehasseb et al., 2010). In the AM global single-cell ecosystem, the components of the SMC are more prominent in the AM than in the controls due to AM-induced SMC abnormalities. SMC clusters were significantly involved in cytokine receptor interactions and vascular smooth muscle contraction. SMCs are capable of significant phenotypic changes in response to changes in local environmental cues, including cell-cell and cell-matrix interactions, as well as various inflammatory mediators (Owens et al., 2004). In addition, vascular smooth muscle contraction occurs, leading to smooth muscle ischemia and dysmenorrhea in AM patients (Zhai et al., 2020). In GRN, we obtained five co-expression modules and three TFs, including REL, MAZ, and ETV7. Studies have demonstrated that REL was expressed and localized in the epithelial or stromal cells after castrated prostate patients (Rosa-Ribeiro et al., 2014). MAZ, as a transcriptional activator, may participate in the development of atherosclerosis (Ponnusamy et al., 2015). ETV7 promotes the resistance of breast cancer cells to chemotherapy and radiotherapy (Pezze et al., 2021). However, these transcription factors have hardly been reported in AM. Generally speaking, maintaining the microenvironment homeostasis of SMC is critical for inhibition of development in AM.

In the present study, we found expression profiles of 42,260 cells and identified 10 cell clusters, including EC, fibroblasts, Ep, En, CD8^+^T, CD4^+^T, Naive T, Mac, pDC, SMC, and ILC. Among there, significant abundance of EC, Ep, En, and SMC in AM patients comparing the controls. Furthermore, the Ep clusters were mainly involved in leukocyte transendothelial cell migration and apoptosis; En clusters were mainly involved in pathways in cancer and apoptosis. Especially, some cell clusters were involved in cell migration and apoptosis may be promote the development of AM patients. Moreover, we obtained co-expression modules and TFs associated with the significant cell clusters by GRN comparing with the previous studies.

In conclusion, our study established a single-cell ecological landscape of the endometrium between control and AM patients, and explored the dynamic changes of immune cells during AM. However, this study has several limitations. First, the samples were too small and larger sample size is needed for a large-scale study. Moreover, this study was mainly based on bioinformatics analysis and therefore requires relevant molecular and cellular experimental validation.

## Data Availability

The datasets presented in this study can be found in online repositories. The names of the repository/repositories and accession number(s) can be found in the article/Supplementary Material.
